# The Harmful Cyanobacterium *Microcystis aeruginosa* Differently Affects the Growth Rate and Photosynthetic Efficiency of Several Species of Marine Phytoplankton

**DOI:** 10.1111/1758-2229.70091

**Published:** 2025-03-28

**Authors:** Na Yun Park, Hyun Soo Choi, Sang Uk Kang, An Suk Lim

**Affiliations:** ^1^ Division of Applied Life Science Gyeongsang National University Jinju Republic of Korea; ^2^ Division of Life Science Gyeongsang National University Jinju Republic of Korea

**Keywords:** cyanobacterial harmful algal bloom, growth inhibition, metabolites, pulse‐amplitude modulated fluorescence

## Abstract

*Microcystis aeruginosa* is a major cyanobacterium that can secrete toxins, such as microcystin, and causes harmful algal blooms. Despite extensive research on the effects of microcystins on other organisms, research on how introduced *M. aeruginosa* into estuaries affects marine phytoplankton is scarce. In this study, the effect of *M. aeruginosa* on the growth of seven representative marine phytoplankton species that inhabit estuaries was determined. The marine phytoplankton species differed in their responses to *M. aeruginosa* ; of the seven species, the growth rate of *Akashiwo sanguinea* was the most affected by *M. aeruginosa* cells and filtrate. Moreover, our results revealed that *M. aeruginosa* affected the growth rate and photosynthetic efficiency of *A. sanguinea* in a density‐ and time‐dependent manner. Our findings suggest that *M. aeruginosa* may alter the phytoplankton community structure in estuarine ecosystems.

## Graphical abstract

The growth rates of some marine phytoplankton commonly found in estuaries were affected by *Microcystis aeruginosa* . Among them, *Akashiwo sanguinea*, which was most impacted, experienced varying effects depending on the exposure time and concentrations of *M. aeruginosa* cells. Our results suggest that introduced *M. aeruginosa* blooms into an estuary may have considerable impacts on the estuarine phytoplankton community.

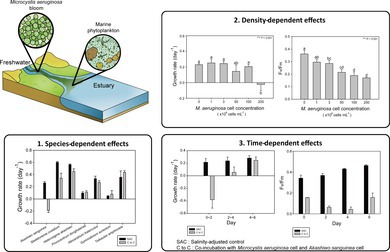

## 1 Introduction

Harmful algal blooms (HABs) are characterised by the excessive growth of phytoplankton, such as cyanobacteria, dinoflagellates and diatoms (Zingone and Enevoldsen 2000). In particular, the increased prevalence of cyanobacterial HABs (cyanoHABs) due to climate change has emerged as a societal concern (O'Neil et al. 2012; Paerl 2016; Wells et al. 2020). CyanoHABs pose a direct risk to human health; exposure to cyanoHABs through recreational activities and consumption of drinking water contaminated by cyanoHABs causes respiratory illness and liver damage, respectively (Falconer et al. 1983; Stewart et al. 2006; Zhang, Lee et al. 2015). As cyanobacteria have a low nutritional value, their proliferation during cyanoHABs results in the loss of high‐quality food resources for zooplankton (Ger et al. 2016), consequently affecting the growth of fish larvae that feed on zooplankton (Karjalainen et al. 2005). Moreover, cyanoHABs are toxic to animals, as evidenced by documented instances of multiple animal fatalities (Wood 2016). In addition, cyanotoxins accumulate within plant tissues, resulting in stunted crop growth (Babica et al. 2006) and reduced yield (Liang et al. 2016). As climate change promotes the proliferation of cyanobacteria (Paerl and Huisman 2009; Kramer et al. 2024), the social and ecological challenges posed by cyanoHABs may further intensify in the future.

*Microcystis aeruginosa* is a freshwater cyanobacterium that is primarily responsible for the occurrence of cyanoHABs (Cook et al. 2020). It is widely distributed in various environments (Zurawell et al. 2005; Harke et al. 2016). *M. aeruginosa* can produce microcystins (Carmichael and Boyer 2016) and lipopolysaccharides (Mayer et al. 2011). The large‐scale *Microcystis* bloom population originating in the river or a lake eventually flows into the estuary. Particularly if the rivers are enormous and flow very slowly with some weir gates in the river, like in Korea, it is common to form large *Microcystis* blooms in the river. This inflow of *M. aeruginosa* bloom can occur actively through increases in precipitation and runoff, affecting marine community (Preece et al. 2017). Freshwater‐derived *M. aeruginosa* has been frequently detected in coastal waters, where it encounters other marine phytoplankton (Jeong et al. 2013; Yoo et al. 2016; Preece et al. 2017; Bormans et al. 2020); for instance, a high density of *Microcystis* species has been frequently observed in the Nakdong River Estuary (Figure S1), where salinity levels fluctuate between 8 and 31 (Yoo et al. 2016). The density of *Microcystis*‐dominated communities introduced into estuarine and marine environments can reach up to 10^5^ cells mL^−1^ (Bormans et al. 2020; Kim, Kim et al. 2021). Hence, it is crucial to examine how marine phytoplankton are affected by the introduction of *M. aeruginosa* into estuaries; however, research focusing on this topic is limited.

As primary producers, phytoplankton contribute to more than 30% of global primary production and play a vital role in supporting ecosystems (Banse 1992; Mulholland and Lomas 2008; Lim et al. 2022). Any disruption in the growth and photosynthesis of phytoplankton may alter marine communities and trigger ripple effects throughout the marine ecosystem. Previous studies have assessed whether phytoplankton are affected by environmental stressors by employing pulse‐amplitude modulated (PAM) fluorescence, which is a well‐established method for detecting photosynthesis inhibition because of allelopathy (Krause and Weis 1984; Chia and Bittencourt‐Oliveira 2021). Changes in the photosynthetic activity of marine phytoplankton caused by *M. aeruginosa* could be measured using PAM, along with assessing the growth rates of phytoplankton.

This study sought to elucidate the potential impacts of the massive blooms of *Microcystis* introduced into estuarine environments, where *Microcystis* and marine phytoplankton may encounter and interact. First, the study aimed to determine the effects of high *M. aeruginosa* concentrations on the growth of seven phytoplankton species (namely, *Akashiwo sanguinea*, *Prorocentrum donghaiense*, *Gymnodinium aureolum*, *Alexandrium fraterculus*, *Heterosigma akashiwo*, *Skeletonema costatum* and *Teleaulax amphioxeia*) that represent different groups of marine phytoplankton and inhabit the estuaries. In addition, the mechanisms behind the growth inhibition of marine phytoplankton caused by *M. aeruginosa* were unravelled by co‐incubating *A. sanguinea* with different concentrations of *M. aeruginosa* cells for an extended period. The findings of this study can contribute valuable insights into the often‐overlooked the effect of introduced *M. aeruginosa* into estuarine environments on the other phytoplankton, elucidate the potential ecological implications, and serve as a foundation for informed environmental management strategies to control the proliferation of *M. aeruginosa* .

## 2 Materials and Methods

### 2.1 Culture of Cyanobacterium and Marine Phytoplankton

The *M. aeruginosa* strain (FBCC‐A60) was obtained from the Nakdong River National Institute of Biological Resources in the Republic of Korea. *M. aeruginosa* culture was incubated in a freshwater‐based BG11 medium and confirmed as a toxic unicellular strain (microcystins 0.1 fg cell^−1^; our unpublished data). *Akashiwo sanguinea* (SA200604), *G. aureolum* (GJ210513), *P. donghaiense* (MS220601), *H. akashiwo* (HAKS01), *T. amphioxeia* (CR‐MAL01), *A. fraterculus* (AFYS1309) and *S. costatum* (SCYS1801) were isolated from the coastal waters of South Korea and established as unialgal cultures, though not free of bacteria. The phytoplankton cultures were incubated in an F/2 medium (Sigma‐Aldrich; Bavaria, Germany) prepared with sterilised seawater, with a salinity range of 34.2–34.8, with the exception of *A. fraterculus* , which was incubated in a seawater‐based L1 medium (Guillard and Hargraves 1993). All cultures were maintained at 20°C ± 2°C under a light intensity of 50 μmol photons m^−2^ s^−1^ and a 14 h:10 h light:dark cycle starting at 8 AM. The phytoplankton cultures used in this experiment were in the exponential growth stage. To maximise the effect of *M. aeruginosa* cells or metabolites on the culture filtrate, the *M. aeruginosa* cells at the stationary stage of development were used in the experiments.

### 2.2 Determining the Effects of *M. aeruginosa* on Various Marine Phytoplankton Species

**FIGURE 1:**
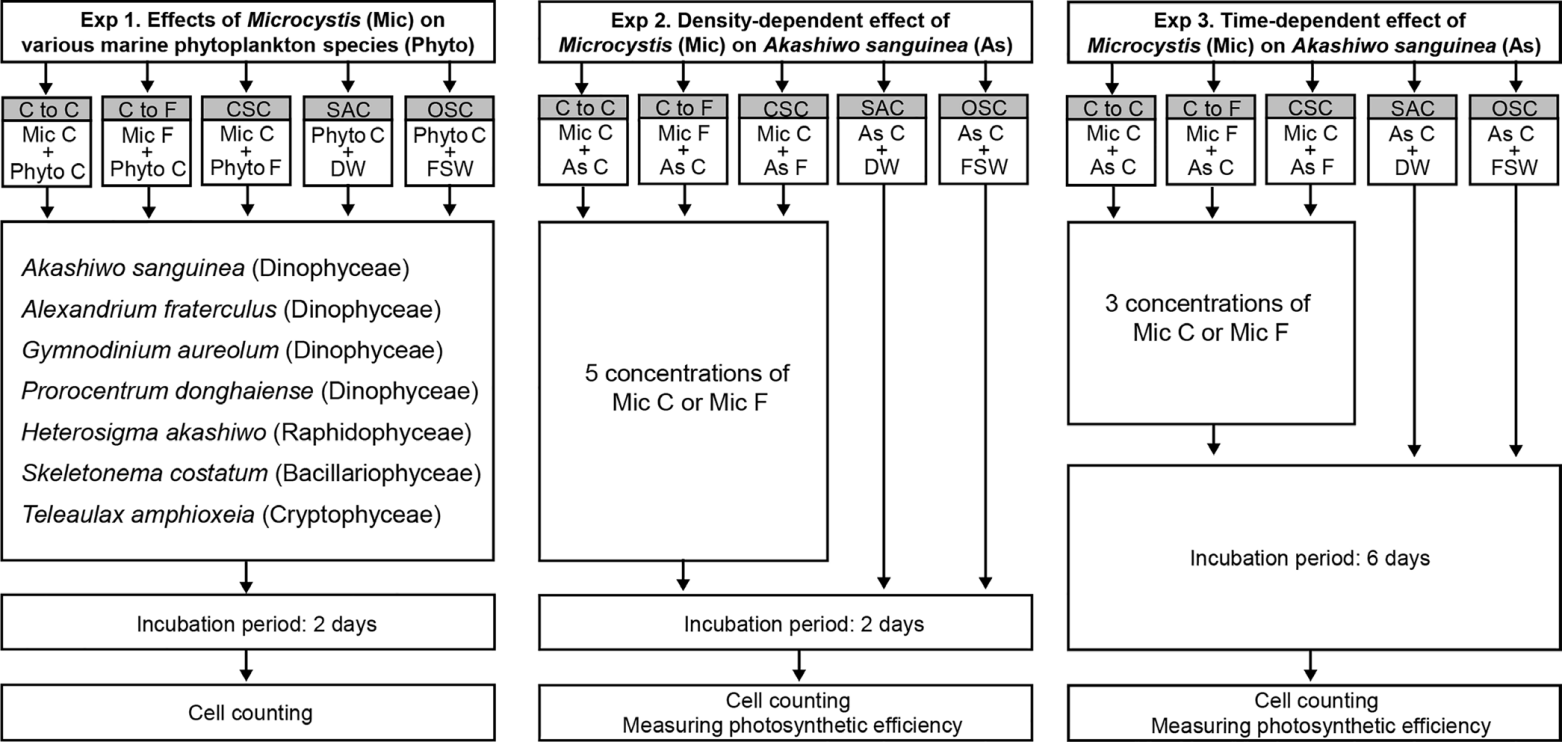
Schematic procedure for Experiments 1–3. C, cells; F, filtrates; CSC, control for making a similar condition to the other experimental treatment; DW, distilled water; FSW, filtered seawater; OSC, original salinity control without distilled water inputs for salinity adjustment. SAC, salinity‐adjusted control, that is, distilled water added to equalise the salinity in the experimental bottles.

Experiment 1 (Exp 1) was conducted to determine whether *M. aeruginosa* affected the growth rate of the various marine phytoplankton by co‐incubating each phytoplankton species with *M. aeruginosa* cells and their filtrate (Figure 1 and Table S1). The seven phytoplankton species represented different groups of marine phytoplankton (Dinophyceae, Raphidophyceae, Bacillariophyceae and Cryptophyceae); they were chosen because they are commonly found in estuaries or form blooms on the coastline and their concentrations often reached up to several 10^4^ cells mL^−1^ (Park et al. 2013; Yoo et al. 2016; Jeong et al. 2017; Lim et al. 2020). The experiment was performed at 20°C in order to compare the physiological responses of marine phytoplankton obtained from the present study to the results of previously published studies. The initial concentrations of each phytoplankton species included in this study were similar to each other in terms of carbon biomass, according to Jeong et al. (2016) and Lim et al. (2018).

Dense cultures of *M. aeruginosa* and each of the marine phytoplankton species growing at 20°C and under a 14:10 h light:dark cycle at 50 μmol photons m^−2^ s^−1^ were transferred into separate 500‐mL polycarbonate (PC) bottles containing F/2 medium. Thereafter, three 1‐mL aliquots were removed from each bottle and enumerated in a Sedgewick–Rafter counting (SRC) chamber under a light microscope (ECLIPSE Ti2; Nikon, Tokyo, Japan) after fixation with 5% Lugol's solution (v/v) to determine the cell density of the aliquots.

**FIGURE 2:**
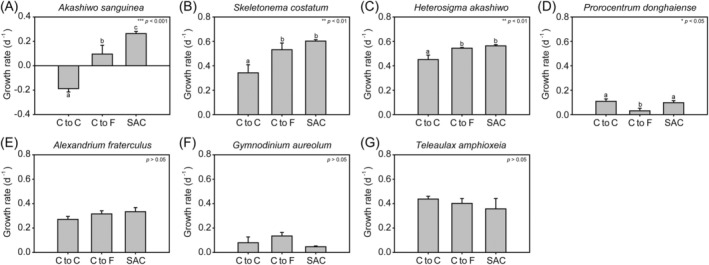
Growth rate of seven marine phytoplankton species co‐incubated with *M. aeroginosa* cell (cells to cells; C to C) and filtrate (cells to filtrates; C to F). (A) *Akashiwo sanguinea*, (B) *Skeletonema costatum*, (C) *Heterosigma akashiwo*, (D) *Prorocentrum donghaiense*, (E) *Alexandrium fraterculus*, (F) *Gymnodinium aureolum* and (G) *Teleaulax amphioxeia*. SAC refers to the salinity‐adjusted control. *t*‐test was performed with *n* = 3 for each group. Statistical significance is indicated as follows: *p < 0.05, **p < 0.01, ***p < 0.001.

For the experiment, the initial concentrations of *M. aeruginosa* and the target phytoplankton species were established using an autopipette to deliver a predetermined volume of culture (with predetermined cell densities) into experimental bottles (42‐mL clear PC bottles). The experimental treatments included (1) triplicate bottles containing phytoplankton cells and *M. aeruginosa* cells (C to C treatment in Figure 1) and (2) triplicate bottles containing phytoplankton cells and *M. aeruginosa* filtrate (C to F treatment in Figure 1). (3) Triplicate bottles containing *M. aeruginosa* cells and each marine phytoplankton filtrate (control for making conditions similar to those seen in the other experimental treatment, CSC in Figure 1) and (4) triplicate bottles containing phytoplankton cells and distilled water (salinity‐adjusted control, SAC, Figure 1) were also prepared. To eliminate the confounding effects of salinity adjustments on the growth rate of marine phytoplankton, (5) triplicates of the original salinity control (OSC, containing marine phytoplankton without distilled water inputs for salinity adjustment, Figure 1) were also prepared. To obtain the filtrates of each algal culture, the *M. aeruginosa* and phytoplankton cultures were filtered through a bottle‐top vacuum filter with a polyethersulfone membrane (pore size: 0.22 μm) or a syringe filter with a surfactant‐free cellulose acetate membrane (pore size: 0.20 μm). The filtrates were added to the C to F treatment bottles in the same volume as the culture added into the C to C treatment bottles for each combination. To prevent the possible effects of different media on the cells, 5 mL of F/2 and saline BG11 each were added to both experimental and control bottles, including SAC and OSC. The final salinity in each bottle was adjusted by adding sterilised and filtered distilled water or seawater to obtain similar water conditions. The bottles were then filled with freshly filtered seawater to reach the target concentration for each algal culture.

To determine the actual algal species concentrations at the beginning of the experiment and after 2 days, 5‐mL aliquots were obtained from each bottle and fixed with 5% Lugol's solution; at least 300 algal cells were enumerated in an SRC chamber. After sub‐sampling, the bottles were refilled with freshly filtered seawater. All experimental bottles were incubated on a 0.9‐rpm rotating wheel under the same light condition and the same temperature as described above. The dilution of the cultures associated with refilling the bottles was considered when calculating the growth rate. The salinity in each bottle was measured after sub‐sampling at the end of the experiment to confirm that all experimental bottles were in the same condition.

The specific growth rate of each phytoplankton cell was calculated as follows:

(1) $$\text{Growth rate}\;\left(d^{-1}\right)=\ln\left(C_{2}/C_{0}\right)/\left(t_{2}-t_{0}\right)$$

where *C*_0_ and *C*_2_ are the cell concentrations (cells mL^−1^) at time points *t*_0_ and *t*_2_, respectively, and *t* represents the time (days).

### 2.3 Effects of *M. aeruginosa* Cell Concentration  and Equivalent Culture Filtrate on  the Growth Rate and Photosynthetic Efficiency of *A. sanguinea*

Experiment 2 (Exp 2) was conducted to determine the density‐dependent effects of different concentrations of *M. aeruginosa* cells and equivalent culture filtrate on the growth rate and photosynthetic efficiency of *A. sanguinea* . *Akashiwo sanguinea* was selected for this experiment because its growth rate was the most affected among the tested marine phytoplankton species in Exp 1. Five different concentrations of *M. aeruginosa* cells (for treatment C to C) and their corresponding culture filtrate (for treatment C to F) were prepared, along with SAC, which was representative of the zero density of *M. aeruginosa* cells and equivalent culture filtrate (Figure 1 and Table S1). To ensure similar water conditions, CSC bottles were prepared by adding identical amounts of *A. sanguinea* culture filtrates to experimental bottles. Additionally, OSC bottles were set up to explore the effect of a change in salinity on *A. sanguinea* during the experiment (Figure 1). Each treatment was performed in triplicate. To obtain the filtrate of *M. aeruginosa* and *A. sanguinea* , each culture was gently filtered through a membrane filter, as described in Section 2.2. The initial concentrations of *M. aeruginosa* cells, *M. aeruginosa* culture filtrate and *A. sanguinea* were prepared using an autopipette to dispense predetermined volumes of culture with known densities into the treatment bottles, as mentioned in Section 2.2 (Table S1). To equalise the salinity of all experimental samples, sterilised distilled water was added to each experimental bottle until the volume of freshwater matched that used in the highest concentration of *M. aeruginosa* .

All experimental bottles were incubated for 2 days under the same conditions as described in Section 2.2. The abundance of *M. aeruginosa* and *A. sanguinea* was measured at the beginning and end of the experiment to calculate the growth rate of the *A. sanguinea* culture. Additionally, the salinity in each bottle was measured at the end of the experiment to confirm that the salinity in all experimental bottles except OSC was similar.

In Exp 1, the growth rate of *A. sanguinea* was reduced by the effect of *M. aeruginosa* cells and their filtrates. To examine whether *M. aeruginosa* affected the photosynthetic efficiency of *A. sanguinea* , the maximum photochemical quantum yield of photosystem II (*F*_*v*_/*F*_*m*_) in *A. sanguinea* was measured using a Phyto PAM II Phytoplankton & Photosynthesis Analyzer (WALZ, Effeltrich, Germany). After sub‐sampling at the end of this experiment, a 4‐mL sample was collected from each bottle and then subjected to photosynthetic efficiency measurement. Prior to the measurement, the samples were kept in the dark for 10 min to relax the reaction centres of photosystem II. Phyto PAM II provides group‐specific values based on multiple wavelengths; thus, the values for *M. aeruginosa* and *A. sanguinea* are distinguishable (Jakob et al. 2005; Lürling et al. 2018). The *F*_*v*_/*F*_*m*_ ratio was measured using a saturation pulse and calculated using the following equations:

(2) $$F_{v}=F_{m}-F_{0}$$

(3) $$F_{v}/F_{m}=\left(F_{m}-F_{0}\right)/F_{m}$$

where *F*_*m*_ is the maximum fluorescence level elicited by a saturation pulse, *F*_0_ is the minimum fluorescence level excited by the very low‐intensity measured light (Kitajima and Butler 1975). The photosynthetic efficiency was monitored using the maximum quantum yield of PSII (*F*_*v*_/*F*_*m*_) as a proxy for the physiological state.

### 2.4 Effects of Prolonged Exposure to *M. aeruginosa* Cells and Equivalent Culture Filtrate on the Growth Rate and Photosynthetic Efficiency of *A. sanguinea*

Experiment 3 (Exp 3) was designed to explore whether prolonged exposure time to *M. aeruginosa* cells and equivalent culture filtrate affects the growth rate and photosynthetic efficiency of *A. sanguinea*. In Exp 2, the photosynthetic efficiency of *A. sanguinea* was significantly inhibited by *M. aeruginosa* cells at concentrations of 3–50 × 10^4^ cells mL^−1^; however, its growth was not inhibited. Hence, we aimed to determine whether the reduced photosynthetic efficiency of *A. sanguinea* would cause a decrease in its growth rate over a prolonged exposure to *M. aeruginosa* (time‐dependent effect). For this, *A. sanguinea* cells were co‐incubated with *M. aeruginosa* for 6 days at a concentration of 3 × 10^5^ cells mL^−1^. Moreover, to investigate whether *A. sanguinea* cells exhibit similar physiological effects when exposed to *M. aeruginosa* cells and filtrates over a prolonged period (6 days), *A. sanguinea* cells were co‐incubated with two different concentrations of *M. aeruginosa* cells and filtrates: (1) 1 × 10^4^ (no changes in photosynthetic efficiency and growth rate of *A. sanguinea* were observed in Exp 2) and (2) 2 × 10^6^ cells mL^−1^ (both photosynthetic efficiency and growth rate of *A. sanguinea* were significantly inhibited in Exp 2) (Figure 1 and Table S1).

Dense cultures of *M. aeruginosa* and *A. sanguinea* growing under the same light and temperature conditions were enumerated under a light microscope as described above to determine each cell concentration and calculate the amount of culture volume to reach the target initial concentration. All samples were incubated in 250‐mL cell culture flasks; filtered seawater was added to reach a total culture volume of 100 mL in the culture flasks. Cell culture filtration and salinity adjustments were performed as described above. In each experimental flask, including CSC, SAC and OSC, 10 mL of F/2 and BG11 media made with seawater were added to prevent nutrient limitation during the incubation and to create similar water conditions in every treatment. All experiments were conducted in triplicate (*n* = 3) and incubated under a light intensity of 50 μmol photons m^−2^ s^−1^ (14 h:10 h light:dark cycle) at 20°C to compare the results obtained from the present study with those from previous literature. Sub‐sampling and measurements of growth rate and photosynthetic efficiency were conducted every 2 days for a total of 6 days, as described above.

### 2.5 Statistical Analysis

The data obtained from the triplicates were checked for normality and homogeneity by performing Shapiro–Wilk test and Levene's tests using the ‘stats’ and ‘car’ packages, respectively. Analysis of variance (ANOVA) or Welch's ANOVA—using ‘Anova Tables’ and ‘stats’ packages, respectively—was performed to test the effects of *M. aeruginosa* concentration and exposure time on the growth rate and photosynthetic efficiency of *A. sanguinea* . Moreover, two‐way ANOVA using the ‘stats’ and ‘lsr’ packages was performed to explore whether the density of *M. aeruginosa* (cells and filtrates) and exposure time to *M. aeruginosa* have an interactive effect in Exp 3. A Bonferroni correction was performed for the two‐way ANOVA tests to correct for multiple testing using the ‘stats’ package. When significant differences were found between treatment groups, post hoc Scheffer or Games‐Howell tests were performed using the ‘agricolae’ and ‘rstartix’ packages, respectively, to identify the groups responsible for such significant differences. An independent *t*‐test was performed to confirm whether salinity adjustments in the experimental bottles except the OSC affected the growth rate and photosynthetic efficiency of marine phytoplankton. For data analysis, R version 4.3.3. (R Core Team 2022) was used.

## 3 Results

### 3.1 Effects of *M. aeruginosa* on Various Marine Phytoplankton

The marine phytoplankton species responded differently to high concentrations of *M. aeruginosa* . Of the seven species tested, the growth rates of three species— *A. sanguinea* , *S. costatum* and *H. akashiwo—*were significantly inhibited by *M. aeruginosa* cells (Figure 2). The growth rate of *A. sanguinea* when co‐incubated with *M. aeruginosa* cells (−0.19 ± 0.03 d^−1^, average ± standard error, SE) was significantly lower than when it was co‐incubated with *M. aeruginosa* filtrate (0.10 ± 0.09 d^−1^); it was also lower than that of the salinity‐adjusted control (SAC, 0.26 ± 0.02 d^−1^) (ANOVA, *F* = 49.309, *p* = 0.000; Figure 2A). The growth rates of *S. costatum* and *H. akashiwo* when co‐incubated with *M. aeruginosa* cells (0.34 ± 0.08 d^−1^ and 0.45 ± 0.05 d^−1^, respectively) were also significantly lower than those when co‐incubated with *M. aeruginosa* filtrate (0.53 ± 0.07 d^−1^ and 0.54 ± 0.01 d^−1^, respectively) as well as those in the SAC (0.60 ± 0.01 d^−1^ and 0.56 ± 0.01 d^−1^, respectively) (ANOVA, *F* = 14.273, *p* = 0.005 and *F* = 14.418, *p* = 0.005, respectively; Figure 2B,C). The growth rate of *P. donghaiense* when co‐incubated with *M. aeruginosa* filtrate (0.03 ± 0.03 d^−1^) was slightly lower than that when co‐incubated with *M. aeruginosa* cells (0.11 ± 0.02 d^−1^) as well as that in the SAC (0.10 ± 0.02 d^−1^) (ANOVA, *F* = 9.348, *p* = 0.014; Figure 2D). *A. fraterculus* , *G. aureolum* and *T. amphioxeia* were not significantly affected by *M. aeruginosa* cells or their filtrate (Figure 2E–G).

In the experimental bottles and SAC, salinity decreased due to the addition of *M. aeruginosa* cultures (range: 27.7–28.6). To determine the effect of reduced salinity on each phytoplankton species, the algal growth rates in SAC and OSC were compared (Figure S2). Even though the growth rate of *H. akashiwo* in the SAC (0.56 ± 0.01 d^−1^) was significantly higher than that in the OSC (0.45 ± 0.01 d^−1^) (*t*‐test, *p* = 0.000), there were no significant differences in the growth rates of other phytoplankton species between the SAC and OSC (*t*‐test, *p* > 0.05; Figure S2).

### 3.2 Effects of Various Concentrations of *M. aeruginosa* Cells and Equivalent Culture Filtrate on the Growth Rate and Photosynthetic Efficiency of *A. sanguinea*

**FIGURE 3:**
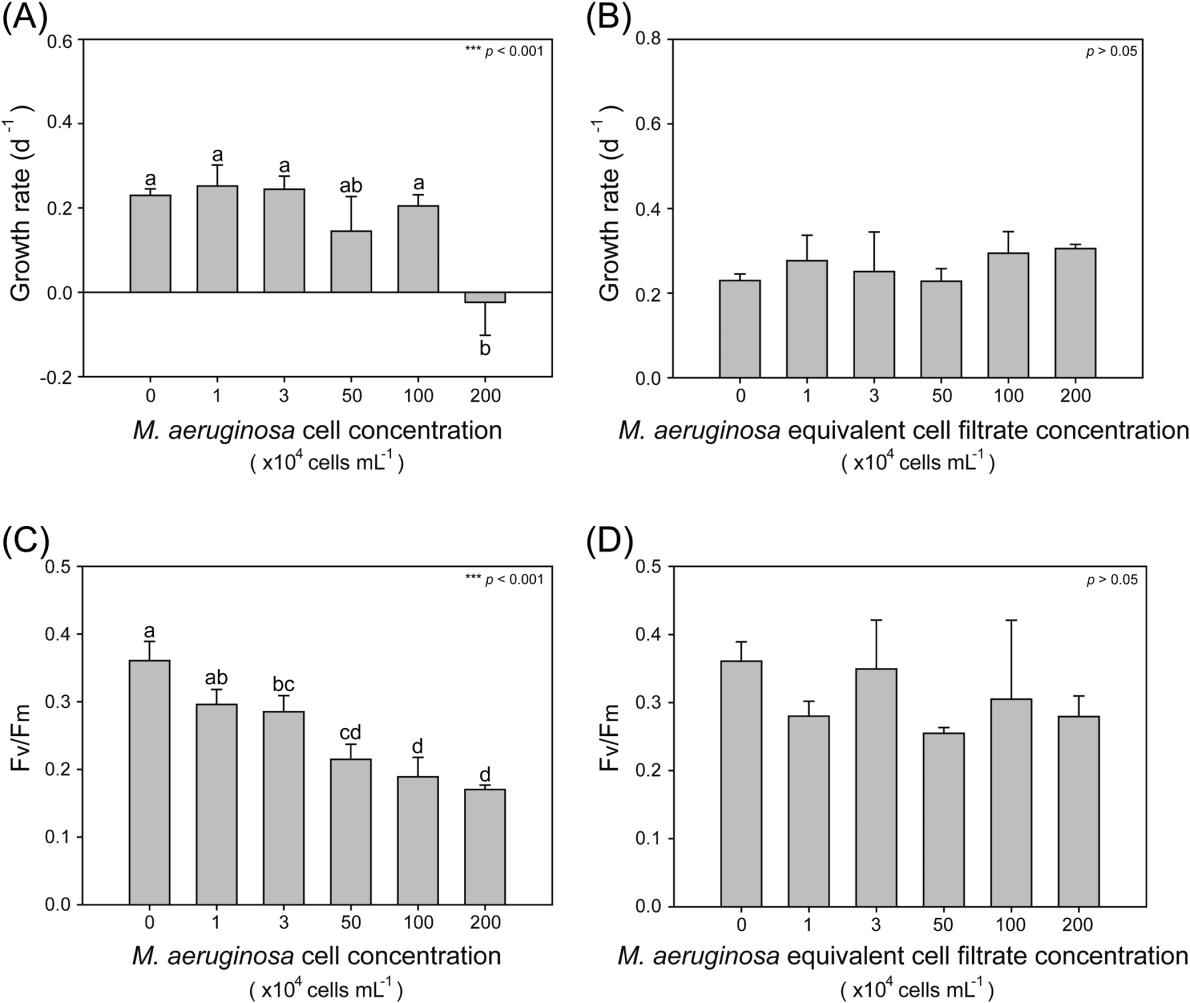
Growth rate (A, B) and photosynthetic efficiency (C, D) of *Akashiwo sanguinea* co‐incubated with various concentrations of *Microcystis aeruginosa* cells (A, C) and the equivalent filtrate (B, D). ANOVA analysis was performed with *n* = 3 for each group. Statistical significance is indicated as follows: ***p < 0.001.

The specific growth rate of *A. sanguinea* was negative at a high *M. aeruginosa* cell concentration of 2 × 10^6^ cells mL^−1^, whereas those at the lower concentrations were positive (Figure 3A). However, there was no significant change in the growth rate of *A. sanguinea* when co‐incubated with various concentrations of *M. aeruginosa* filtrate (ANOVA, *p* > 0.05; Figure 3B).

The photosynthetic efficiency (*F*_*v*_/*F*_*m*_) of *A. sanguinea* was inhibited at concentrations higher than 1 × 10^4^ cells mL^−1^ of *M. aeruginosa* cells (Figure 3C). The mean *F*_*v*_/*F*_*m*_ values (0.17–0.29) of *A. sanguinea* co‐incubated with *M. aeruginosa* cells at concentrations ranging from 3 × 10^4^ to 2 × 10^6^ cells mL^−1^ were significantly lower than that without *M. aeruginosa* cells (0.36 ± 0.03) (ANOVA, *F* = 29.899, *p* = 0.000; Figure 3C). Although the photosynthetic efficiency of *A. sanguinea* decreased at *M. aeruginosa* cell concentrations ranging from 3 × 10^4^ to 100 × 10^4^ cells mL^−1^, its growth rate was unaffected (Figure 3A,C). Similarly, the *M. aeruginosa* filtrate did not inhibit the photosynthetic efficiency of *A. sanguinea* (ANOVA, *p* > 0.05; Figure 3D).

The growth rate of *A. sanguinea* in the SAC (0.23 ± 0.02 d^−1^) was not significantly different from that in the OSC (0.24 ± 0.05 d^−1^) (*t*‐test, *p* > 0.05; Figure S3). The photosynthetic efficiency of *A. sanguinea* in the SAC (0.36 ± 0.03 d^−1^) was slightly higher than that in the OSC (0.28 ± 0.02 d^−1^) (*t*‐test, *p* < 0.05; Figure S3). The salinity in the experimental and SAC bottles ranged between 28.3–29.3, whereas that in OSC bottles ranged between 34.2 and 34.4.

### 3.3 Effects of Exposure Time to *M. aeruginosa* Cells and Equivalent Culture Filtrate on the Growth Rate and Photosynthetic Efficiency of *A. sanguinea*

**FIGURE 4:**
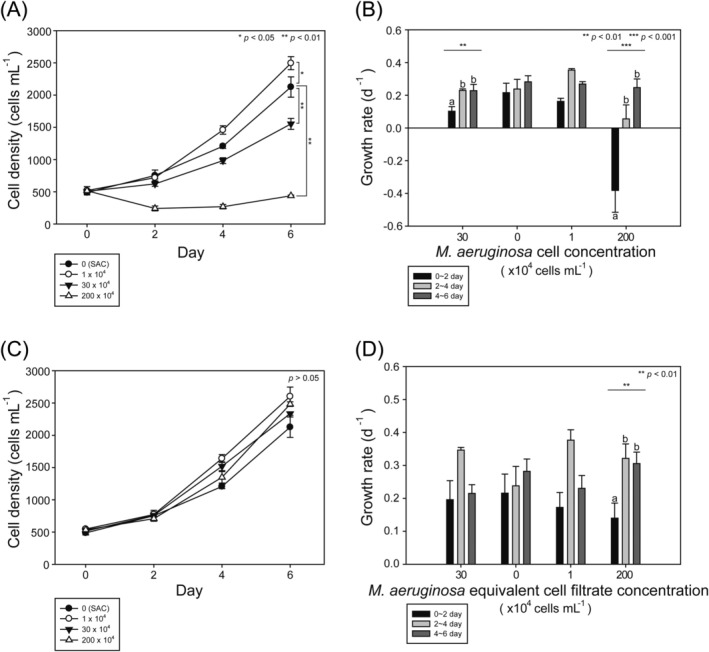
Change in cell density (A, C) and growth rate (B, D) of *Akashiwo sanguinea* co‐incubated with three different concentrations of *Microcystis aeruginosa* cells (A, B) and the equivalent filtrate (C, D) for 6 days. SAC, salinity‐adjusted control. *t*‐test and ANOVA analyses were performed with *n* = 3 for each group. Statistical significance is indicated as follows: **p < 0.01, ***p < 0.001.

When *A. sanguinea* cells were exposed to 30 × 10^4^ cells mL^−1^ of *M. aeruginosa* cells for an extended experimental period, the number of *A. sanguinea* cells slowly increased (Figure 4A). The growth rate of *A. sanguinea* , however, was slightly inhibited by *M. aeruginosa* cells for the first 2 days but slowly recovered as incubation progressed (ANOVA, *F* = 12.958, *p* = 0.009; Figure 4B). When co‐incubated with other concentrations of *M. aeruginosa* cells (from 0 cells mL^−1^ in SAC to the highest concentration of 200 × 10^4^ cells mL^−1^), *A. sanguinea* cells exhibited different growth patterns with an increase in exposure time to *M. aeruginosa* cells (Figure 4A). At zero to low concentrations of *M. aeruginosa* cells (0–1 × 10^4^ cells mL^−1^), *A. sanguinea* cells increased in number as time elapsed; their growth rates also slightly fluctuated but maintained positive levels (Figure 4A,B). However, at the highest concentration of *M. aeruginosa* cells (200 × 10^4^ cells mL^−1^), the number of *A. sanguinea* cells significantly decreased for the first 2 days but slightly increased later (Figure 4A). Consequently, the growth rate of *A. sanguinea* at this concentration of *M. aeruginosa* cells was negative during the first 2 days but slowly recovered after 4 days (ANOVA, *F* = 48.113, *p* = 0.000; Figure 4B). On Day 6, although the growth rate of *A. sanguinea* recovered slightly (when co‐incubated with 30 × 10^4^ cells mL^−1^ and 200 × 10^4^ cells mL^−1^ of *M. aeruginosa* cells), the number of cells remained significantly lower than that in the SAC (Welch two‐sample *t*‐test, *p* < 0.01; Figure 4A). The densities and growth rates of *A. sanguinea* at all concentrations of *M. aeruginosa* filtrate were comparable (Figure 4C,D). For the first 2 days, the growth rate of *A. sanguinea* in the SAC (0.22 ± 0.06 d^−1^) was different from that in the OSC (0.06 ± 0.05 d^−1^) (*t*‐test, *p* < 0.05; Figure S4), but these growth rates became similar after 2 days. The salinity in the experimental, CSC and SAC bottles ranged between 28.3 and 28.7 and that in the OSC bottles ranged 34.6–34.8.

**FIGURE 5:**
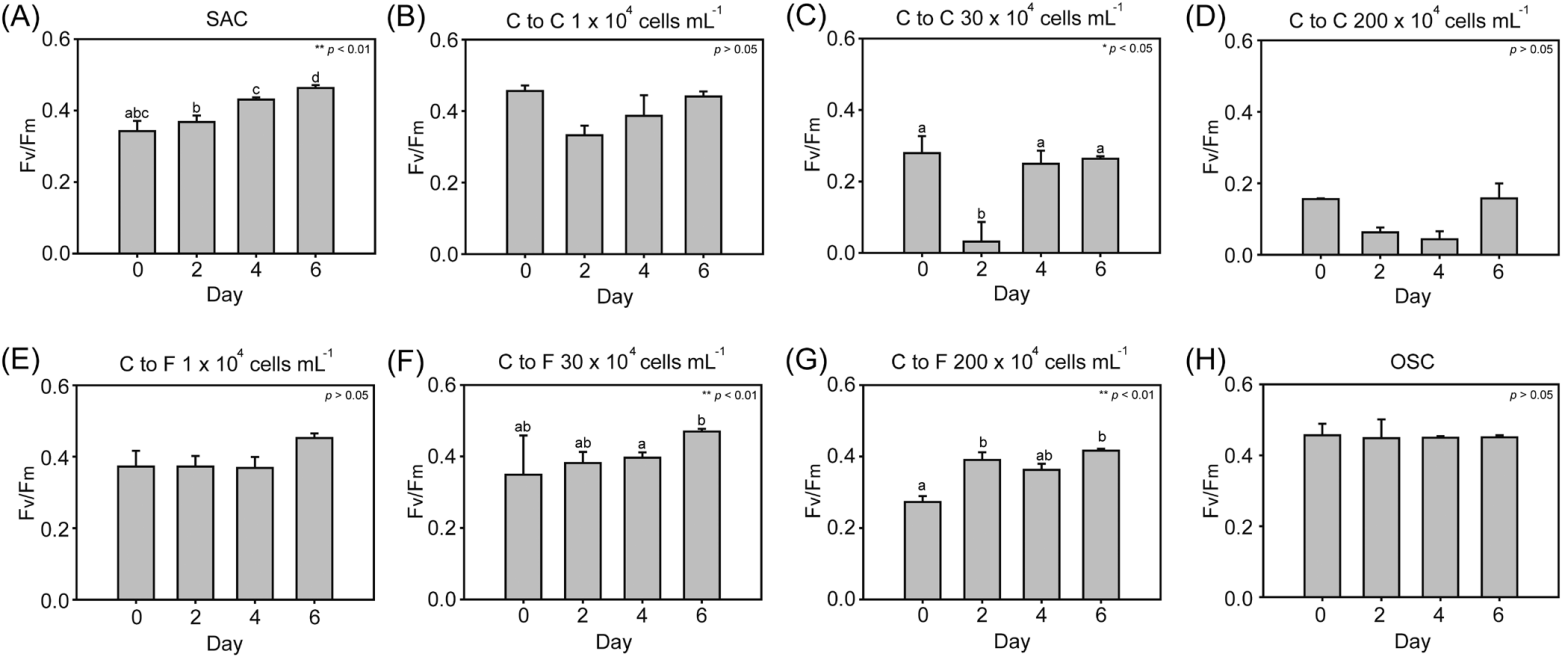
Change in the photosynthetic efficiency of *Akashiwo sanguinea* co‐incubated with various concentrations of *Microcystis aeruginosa* cells and the equivalent filtrate for 6 days. Photosynthetic efficiency when co‐incubated with *Microcystis aeruginosa* cell concentrations of (A) zero cells of *M. aeruginosa* (salinity‐adjusted control, SAC); (B) 1 × 10^4^ cells mL^−1^, (C) 30 × 10^4^ cells mL^−1^, (D) 200 × 10^4^ cells mL^−1^. Photosynthetic efficiency when co‐incubated with *Microcystis aeruginosa* equivalent filtrate concentrations of (E) 1 × 10^4^ cells mL^−1^, (F) 30 × 10^4^ cells mL^−1^, (G) 200 × 10^4^ cells mL^−1^ and (H) original salinity control (OSC). Welch's ANOVA analysis was performed with *n* = 3 for each group. Statistical significance is indicated as follows: *p < 0.05, **p < 0.01.

When considered as a function of exposure time to *M. aeruginosa* cells, the *F*_*v*_/*F*_*m*_ of *A. sanguinea* at the concentration of 30 × 10^4^ cells mL^−1^ *M. aeruginosa* cells significantly decreased after 2 days of incubation, which was expected based on Exp 2 results (Figure 5). However, it recovered with continued exposure by Day 6 (Welch ANOVA, *F* = 12.919, *p* = 0.023). The *F*_*v*_/*F*_*m*_ of *A. sanguinea* in the SAC ranged from 0.34 to 0.46; the value slightly increased during the remaining experimental period (Figure 5). At a low concentration of *M. aeruginosa* cells (1 × 10^4^ cells mL^−1^), the *F*_*v*_/*F*_*m*_ of *A. sanguinea* slightly decreased for the first 2 days and then plateaued for the remaining period; in contrast, at high concentrations of *M. aeruginosa* cells (> 200 × 10^4^ cells mL^−1^), the values were consistently low throughout the 6 days of incubation (Figure 5). When *A. sanguinea* cells were exposed to three different concentrations of *M. aeruginosa* filtrate for 6 days, the mean *F*_*v*_/*F*_*m*_ values ranged from 0.27 to 0.47 (ANOVA, *p* > 0.05, Figure 5E; Welch ANOVA, *F* = 18.929, *p* = 0.009, Figure 5F and ANOVA, *F* = 18.870, *p* = 0.001 for Figure 5G). The *F*_*v*_/*F*_*m*_ of *A. sanguinea* in the OSC was maintained at about 0.46 ± 0.03 (Figure 5H). The photosynthetic efficiency of *A. sanguinea* in the SAC (0.34 ± 0.03) at the beginning of the experiment was slightly lower than that in the OSC (0.46 ± 0.03) (*t*‐test, *p* < 0.05, Figure S5); however, they subsequently exhibited similarities after 2 days. The salinity in the experimental, CSC and SAC bottles ranged 28.3–28.7, and that in the OSC bottles ranged 34.6–34.8.

The two‐way ANOVA results showed an interactive effect between *M. aeruginosa* cells or filtrate concentrations and exposure time on the growth rate of *A. sanguinea* . *M. aeruginosa* cell concentration and exposure time interacted with the growth rate of *A. sanguinea* (two‐way ANOVA, *F* = 17.632, *p* = 0.000). Both *M. aeruginosa* cell concentration (*F* = 48.643, *p* = 0.000) and exposure time (*F* = 56.927, *p* = 0.000) significantly influenced the growth rate of *A. sanguinea* (Figure S6 and Table S2A). Among the three factors, the effect size of *M. aeruginosa* cell concentration was the largest (partial eta squared, *η_p_* ^2^ = 0.859). When *A. sanguinea* cells were co‐incubated with *M. aeruginosa* filtrate, the factors influencing the growth rate of *A. sanguinea* were exposure time (two‐way ANOVA, *F* = 30.730, *p* = 0.000) as well as the interaction between the concentration of *M. aeruginosa* filtrate and the exposure time (two‐way ANOVA, *F* = 4.935, *p* = 0.006), but not *M. aeruginosa* filtrate concentration (two‐way ANOVA, *F* = 0.460, *p* = 1.000). Between the two factors, the effect size of exposure time was more significant than the others (*η_p_* ^2^ = 0.719). When *A. sanguinea* was co‐incubated with *M. aeruginosa* cells, the factors that influenced its *F*_*v*_/*F*_*m*_ included *M. aeruginosa* cell concentration (two‐way ANOVA, *F* = 276.210, *p* = 0.000), exposure time (two‐way ANOVA, *F* = 44.239, *p* = 0.000), and the interactive effect (two‐way ANOVA, *F* = 9.870, *p* = 0.000) (Figure S6 and Table S2B). Among the three factors, the effect size of *M. aeruginosa* cell concentration was the largest (*η_p_* ^2^ = 0.963). However, when *A. sanguinea* cells were co‐incubated with *M. aeruginosa* filtrates, only one factor, exposure time affected the photosynthetic efficiency of *A. sanguinea* (two‐way ANOVA, *F* = 22.634, *p* = 0.000). In this case, no effects were observed for *M. aeruginosa* filtrate concentration (two‐way ANOVA, *F* = 3.502, *p* = 0.081) and the interaction between the *M. aeruginosa* filtrate concentration and the exposure time (two‐way ANOVA, *F* = 1.694, *p* = 0.396).

## 4 Discussion

### 4.1 Effect of *M. aeruginosa* on Seven Marine Phytoplankton Species

Among the seven marine phytoplankton species tested, the growth rates of *A. sanguinea* , *S. costatum* , *H. akashiwo* and *P. donghaiense* were inhibited by *M. aeruginosa* cells and/or *M. aeruginosa* filtrate. The inhibition of algal growth through interspecies interaction can be attributed to two factors: Allelopathic effect by the other species and competition for limited resources in a restricted space (Tilman 1977; Lim et al. 2014; Chia and Bittencourt‐Oliveira 2021). In this study, *M. aeruginosa* was incapable of growth due to high salinity, and sufficient nutrient supply was provided during incubation; thus, the competition for nutrients for growth between *M. aeruginosa* and other marine phytoplankton was relatively negligible. Moreover, the growth rates of phytoplankton in the OSC, containing both BG11 and F/2 media, were comparable with their previously reported growth rates in optimum media (Fu et al. 2008; Jeong et al. 2010; Menden‐Deuer and Montalbano 2015), indicating that changing media had no effect on the results reported in the present study. For example, the growth rates of *A. sanguinea* (0.20–0.24 d^−1^) obtained in our study were within the range of those reported for various strains grown in the F/2 medium (0.10–0.38 d^−1^, Menden‐Deuer and Montalbano 2015). Therefore, the growth inhibition of marine phytoplankton in our study was attributed to have been driven by the allelopathic effect of *M. aeruginosa* .

Previous studies have also reported that *M. aeruginosa* negatively affects the growth rates of some freshwater algae, such as *Planktothrix agardhii* , *Scenedesmus quadricauda* , *Chlorella pyrenoidosa* and *Cyclotella meneghiniana* , via cell‐to‐cell contact (Wang et al. 2017; Briand et al. 2019). Moreover, growth inhibition due to direct cell‐to‐cell interactions has been known to be greater than that due to exposure to filtrate (Uchida et al. 1999; Yamasaki et al. 2011; Lim et al. 2014). Cyanobacteria such as *M. aeruginosa* produce various secondary metabolites, including toxins (Mayer et al. 2011; Carmichael and Boyer 2016). The microcystins released by *M. aeruginosa* can be regarded as allelochemicals because of their ability to reduce the growth of other organisms (Chaïb et al. 2021). When *M. aeruginosa* cells are lysed, they secrete microcystins into their surrounding environment (Kaplan et al. 2016; Du et al. 2017), accumulating allelopathic effects on other coexisting organisms. Although the present study did not provide a direct evidence of the allelopathic effect by detecting microcystins or metabolites secreted by *M. aeruginosa* , there was a high possibility that the metabolites released from lysed *M. aeruginosa* cells due to salinity might have deposited in waters in the experimental bottles and had a stronger growth‐inhibiting effect than those found in the filtrate.

Unlike *A. sanguinea* , *S. costatum* and *H. akashiwo* , some phytoplankton, such as *G. aureolum* and *T. amphioxeia* , were not affected by *M. aeruginosa* cells or filtrate. This suggests that these species may need more time to be influenced by *M. aeruginosa* cells or filtrate compared to the other species that showed the immediate growth inhibition effect observed within 2 days of incubation, or marine phytoplankton might respond differently to *M. aeruginosa* blooms introduced into estuaries. *M. aeruginosa* can affect the growth of estuarine phytoplankton, including *A. sanguinea* , *S. costatum* and *H. akashiwo* and can potentially alter their dynamics when cyanoHABs occur in estuaries.

### 4.2 Density‐ and Time‐Dependent Effects of *M. aeruginosa* Cells and Filtrate on the Growth and Photosynthetic Efficiency of *A. sanguinea*

*M. aeruginosa* affected the growth rate and photosynthetic efficiency of *A. sanguinea* in a density‐dependent manner. When *A. sanguinea* cells were incubated with various concentrations of *M. aeruginosa* cells, the growth rate and photosynthetic efficiency of *A. sanguinea* were significantly reduced for certain concentrations of *M. aeruginosa* cells. In particular, the growth rate of *A. sanguinea* decreased at 200 × 10^4^ cells mL^−1^ of *M. aeruginosa* cells, which is comparable to or higher than the actual *M. aeruginosa* concentration in estuaries during cyanoHABs (6 × 10^4^ to 1 × 10^6^ cells mL^−1^) (Rocha et al. 2002; Taş et al. 2006; Bormans et al. 2020; Kim, Kim et al. 2021). Therefore, *M. aeruginosa* can affect the growth of *A. sanguinea* and can potentially alter the population dynamics of *A. sanguinea* when cyanoHABs occur in estuaries.

Furthermore, *M. aeruginosa* influenced the growth rate and photosynthetic efficiency of *A. sanguinea* in a time‐dependent manner. When *A. sanguinea* cells were incubated with 30 × 10^4^ cells mL^−1^ of *M. aeruginosa* cells for 2 days, the photosynthetic efficiency and growth rate of *A. sanguinea* showed different responses to the exposure of *M. aeruginosa* cells, expecting that the prolonged exposure time to *M. aeruginosa* cells at the same concentration may lead to constant inhibition of the photosynthetic efficiency of *A. sanguinea* and, eventually, cause growth inhibition. Our results from the extended incubation experiment between *M. aeruginosa* and *A. sanguinea* (Exp 3) showed that the inhibited photosynthetic efficiency of *A. sanguinea* by 30 × 10^4^ cells mL^−1^ of *M. aeruginosa* cells resulted in the inhibition of the growth rate of *A. sanguinea* for the first 2 days of incubation, as expected. However, interestingly, the photosynthetic efficiency of *A. sanguinea* recovered after 4 days, and its growth rate subsequently recovered. Similar results were also observed when *A. sanguinea* cells were co‐incubated with a higher concentration of *M. aeruginosa* cells. At 30 × 10^4^ cells mL^−1^ of *M. aeruginosa* , the effect of *M. aeruginosa* was immediate and intense. Thus, the cell density of *A. sanguinea* drastically decreased at high concentrations of *M. aeruginosa* ; however, the growth rate of *A. sanguinea* recovered as the experiment progressed. Notably, as exposure time elapsed, the effect of *M. aeruginosa* on *A. sanguinea* changed. The similarity in the recovery patterns of growth rate and photosynthetic efficiency of *A. sanguinea* may be due to the degradation of microcystins in saline water over time, which is in agreement with the results of a previous study (Qiu et al. 2022).

Two‐way ANOVA result also showed that the concentration of *M. aeruginosa* cells and incubation time had an interactive effect on the growth rate and photosynthetic efficiency of *A. sanguinea* , meaning that the time‐dependent effect of *M. aeruginosa* cells based on the incubation time differed by *M. aeruginosa* cell concentrations. Based on the effect size of each factor, as the cell concentration of *M. aeruginosa* increased, the effect of *M. aeruginosa* cell concentration on the growth rate of *A. sanguinea* became greater. Considering the effect of *M. aeruginosa* filtrate on the growth rate and the photosynthetic efficiency of *A. sanguinea* , the results indicate that the exposure time to the filtrate of *M. aeruginosa* may have a greater effect on the growth rate and photosynthetic efficiency of *A. sanguinea* than *M. aeruginosa* filtrate concentration. Thus, *M. aeruginosa* had a density and time‐dependent effect on the growth rate and the photosynthetic efficiency of *A. sanguinea. Akashiwo sanguinea* causes algal blooms in the coastal waters of various countries, including Korea, the United States, Japan and Ireland, with densities as high as 3000 cells mL^−1^ (Nakamura and Hirata 2006; Badylak et al. 2014; Menden‐Deuer and Montalbano 2015; Lim et al. 2020). Although the extended 6‐day incubation time may not be enough to explore the change in the population dynamics of a specific algal species, the straightforward effects of *M. aeruginosa* on *A. sanguinea* were revealed in the present study. Therefore, if a high density of *M. aeruginosa* is continuously introduced into an estuary, it may affect the bloom dynamics of *A. sanguinea* at least temporarily.

The *F*_*v*_/*F*_*m*_ values have been used as sensitive photosynthetic performance indicators in many microalgae (Geider et al. 1993; Heraud and Beardall 2000). The *F*_*v*_/*F*_*m*_ value of *A. sanguinea* in the OSC (0.31–0.44) was within the range of the *F*_*v*_/*F*_*m*_ values of other dinoflagellate species at similar temperatures (20°C ± 1°C) (Table 1). For example, the *F*_*v*_/*F*_*m*_ of *P. donghaiense* was 0.25, whereas that of *Alexandrium minutum* was 0.76 (Table 1). These *F*_*v*_/*F*_*m*_ values can be lowered when an organism is exposed to stressors such as nutrient depletion, osmotic stress, salinity and allelopathic effects (Le Rouzic 2012; Qi et al. 2013; Liang et al. 2014; Chia and Bittencourt‐Oliveira 2021). In the present study, salinity and allelopathic effects were considered as the possible influencing factors on the *F*_*v*_/*F*_*m*_ values of *A. sanguinea*. However, controls comparison indicated that salinity had no significant impact on the *F*_*v*_/*F*_*m*_ values, and *A. sanguinea* quickly adapted to changes in salinity (Figure 5B,H). Therefore, the possible salinity effect between treatments in the present study could be ruled out. The impact of *M. aeruginosa* cells or filtrates on the *F*_*v*_/*F*_*m*_ of *A. sanguinea* was significant, suggesting that the allelopathy of *M. aeruginosa* may play a key role in the rapid decline of photosynthetic efficiency in *A. sanguinea* during the initial phase of prolonged incubation. The difference in the *F*_*v*_/*F*_*m*_ values at the different *M. aeruginosa* cell concentrations on Day 0 (0.16–0.46, Figure 5) also indicated that the allelopathic effect of *M. aeruginosa* was strong enough to influence *A. sanguinea* immediately during PAM measurement preparation and dark adaptation. *A. sanguinea* is known to exhibit mixotrophy, and the possibility of this grazing on *M. aeruginosa* cannot be excluded (Bockstahler and Coats 1993). However, microscopic observations did not reveal *A. sanguinea* grazing on *M. aeruginosa* (data not shown). Additionally, since *Karlodinium veneficum* maintained high *F*_*v*_/*F*_*m*_ values while grazing on *Rhodomonas salina* , it is unlikely that the mixotrophic behaviour of *A. sanguinea* significantly influenced *F*_*v*_/*F*_*m*_ (Lin and Glibert 2019). Therefore, the allelopathic effect of *M. aeruginosa* may act as a stressor on *A. sanguinea* . This result was further supported by the changes in *A. sanguinea* cell density and *F*_*v*_/*F*_*m*_ over the experimental period. In particular, when the *F*_*v*_/*F*_*m*_ value of *A. sanguinea* decreased, its growth rate also decreased. However, as the *F*_*v*_/*F*_*m*_ of *A. sanguinea* increased over time, its growth rate recovered accordingly. The *F*_*v*_/*F*_*m*_ value provides insights into the intracellular activity of photosystem II (Kitajima and Butler 1975). It is also known that microcystin affects photosynthetic efficiency and energy flow through photosystem II (Perron et al. 2012). As the inhibition of photosystem II efficiency results in the inhibition of the intracellular CO_2_ fixation ability, a decline in photosystem II functionality could potentially impede various essential metabolic pathways crucial for cellular proliferation (Larkum et al. 2020). Thus, measuring *F*_*v*_/*F*_*m*_ to discover biological interactions between microalgae may be worthwhile.

**TABLE 1:** Photosynthetic efficiency (*F*_*v*_/*F*_*m*_) values of various species in the Dinophyceae family.

Species	*F*_*v*_/*F*_*m*_	Reference
*Akashiwo sanguinea*	0.31–0.44	This study
	0.58	Chen et al. (2021)
	0.48–0.50	Ou et al. (2017)
*Prorocentrum donghaiense*	0.65	Zhang et al. (2018)
	0.52	Shi et al. (2017)
	0.40–0.44	Qi et al. (2013)
	0.34	Zhang, Zhang, et al. (2015)
	0.25–0.30	Zhang et al. (2019)
*Prorocentrum minimum*	0.53	Abassi and Ki (2022)
	0.52	Wang et al. (2019)
	0.35–0.46	Kim, Wang et al. (2021)
	0.38–0.47	Kim et al. (2020)
*Prorocentrum shikokuense*	0.55	Zhang et al. (2022)
*Alexandrium minutum*	0.70–0.76	Deschaseaux et al. (2019)
	0.53–0.61	Rahav and Herut (2023)
*Alexandrium tamarense*	0.33–0.37	Qi et al. (2013)
*Alexandrium pacificum*	0.53	Chen et al. (2022)
*Scrippsiella trochoidea*	0.54–0.56	Qi et al. (2013)
*Karlodinium veneficum*	0.53–0.579	Meng et al. (2019)
*Cochlodinium polykrikoides*	0.62	Guo et al. (2016)
	0.56–0.57	Abassi et al. (2017)

Large‐scale blooms of *Microcystis* in rivers are transported to estuaries through river flow, and these freshwater HABs are likely to have significant impacts on estuarine marine communities, affecting not only phytoplankton but also shellfish and mammals (Miller et al. 2010; Preece et al. 2017). When a *Microcystis*‐dominant community reaches an estuary, most *Microcystis* cells undergo lysis because of high salinity (Bormans et al. 2019). During this process, allelochemicals such as microcystins and other secondary molecules can affect marine phytoplankton, as demonstrated in our experimental results. Additionally, some *Microcystis* cells may not be immediately destroyed because of their mucilage (Bormans et al. 2023), allowing them to remain intact in the water column for at least several days. Even if these cells are not in optimal condition, they can still influence the growth and photosynthetic efficiency of surrounding marine phytoplankton. Toxins derived from *Microcystis* have been consistently detected in estuarine and coastal areas in countries like the United States and Korea, and they have even been found within marine organisms because of bioaccumulation (Miller et al. 2010; Kim, Kim et al. 2021). As global warming is anticipated to increase the prevalence of freshwater HABs, understanding their impacts on marine communities, including phytoplankton, is becoming increasingly crucial. Given the potential impact of *M. aeruginosa* blooms on estuarine phytoplankton communities, future strategies for monitoring *M. aeruginosa* proliferation are essential.

## Author Contributions

**Na Yun Park:** data curation, writing – original draft. **Hyun Soo Choi:** conceptualization, visualization, investigation. **Sang Uk Kang:** investigation, visualization. **An Suk Lim:** conceptualization, writing – review and editing, supervision.

## Ethics Statement

The authors have nothing to report.

## Conflicts of Interest

The authors declare no conflicts of interest.

## Supporting information


Data S1.

## Data Availability

The data that support the findings of this study are available on request from the corresponding author. The data are not publicly available due to privacy or ethical restrictions.
