# Supplementary material for: The Harmful Cyanobacterium Microcystis aeruginosa Differently Affects the Growth Rate and Photosynthetic Efficiency of Several Species of Marine Phytoplankton
Source: Environ Microbiol Rep. 2025 Mar 28;17(2):e70091. doi: 10.1111/1758-2229.70091 (PMC11953570; doi:10.1111/1758-2229.70091)
Supplement: Supplementary file 1 — Data S1. [file EMI4-17-e70091-s001.docx]

**Supplementary materials**

**The harmful cyanobacterium Microcystis aeruginosa differently affects the growth rate and photosynthetic efficiency of several species of marine phytoplankton**

Na Yun Park^1^, Hyun Soo Choi^1^, Sang Uk Kang^1^, and An Suk Lim^1,2*^

^1^Division of Applied Life Science, Gyeongsang National University, Jinju 52828, Republic of Korea

^2^Division of Life Science, Gyeongsang National University, Jinju 52828, Republic of Korea

^*^Corresponding author

E-mail address: aslim@gnu.ac.kr

**Results**

**The growth rate and photosynthetic efficiency of *Microcystis aeruginosa***

In experiment 1, the growth rates of *M. aeruginosa* in CSC were negative (−0.07 ± 0.11 d^-1^) during the 2-day incubation period. Furthermore, *M. aeruginosa* cells showed negative growth rates (−0.10 ± 0.11 d^-1^) when co-incubated with each phytoplankton. The mean growth rate of *M. aeruginosa* cells in OSC, however, was 0.06 ± 0.07 d^-1^ (data not shown). The salinities in the experimental bottles and SAC ranged from 27.7 to 28.6 due to the mixing of *M. aeruginosa* and marine phytoplankton cultures.

In experiment 2, when *M. aeruginosa* cells were co-incubated with *A. sanguinea* (C to C) for 2 days, the growth rate of *M. aeruginosa* (−0.09 ± 0.13 d^-1^) was negative. Similarly, the growth rate of *M. aeruginosa* in the CSC was near zero (−0.00 ± 0.09 d^-1^). Meanwhile, the mean growth rate of *M. aeruginosa* in OSC was 0.15 ± 0.04 d^-1^. The F_v_/F_m_ values for *M. aeruginosa* in both experimental (C to C) and control (CSC) bottles on Day 2 ranged from 0.00 to 0.06.

In experiment 3, the density of *M. aeruginosa* in the experimental and CSC bottles gradually decreased during the 6-day incubation period due to high salinities, ranging from 28.3 to 28.7 (Fig. S5). However, the density of *M. aeruginosa* in OSC gradually increased, resulting in high densities (3,592,500–4,142,500 cells mL^-1^) at the end of the incubation (Fig. S5). With the exception of the values on Day 0, the F_v_/F_m_ values of *M. aeruginosa* ranged from 0.00 to 0.03 during the incubation.


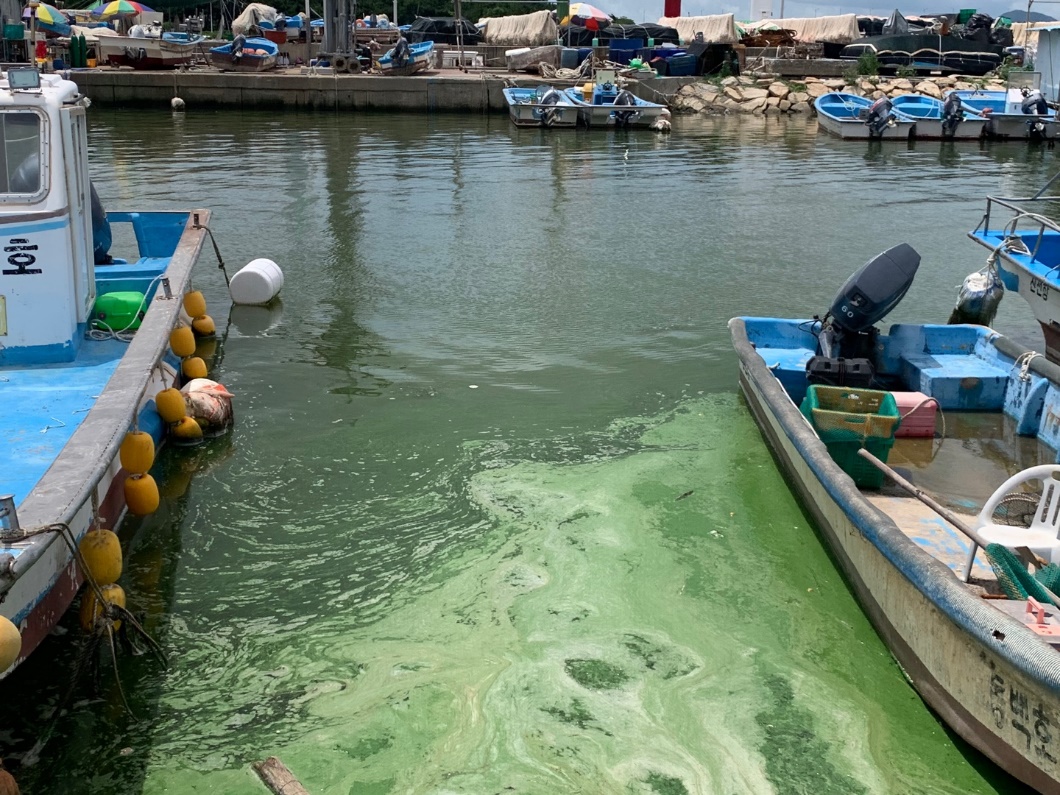
**Supplementary Figure 1.** *Microcystis* sp. flowed down to the Nakdong River Estuary.


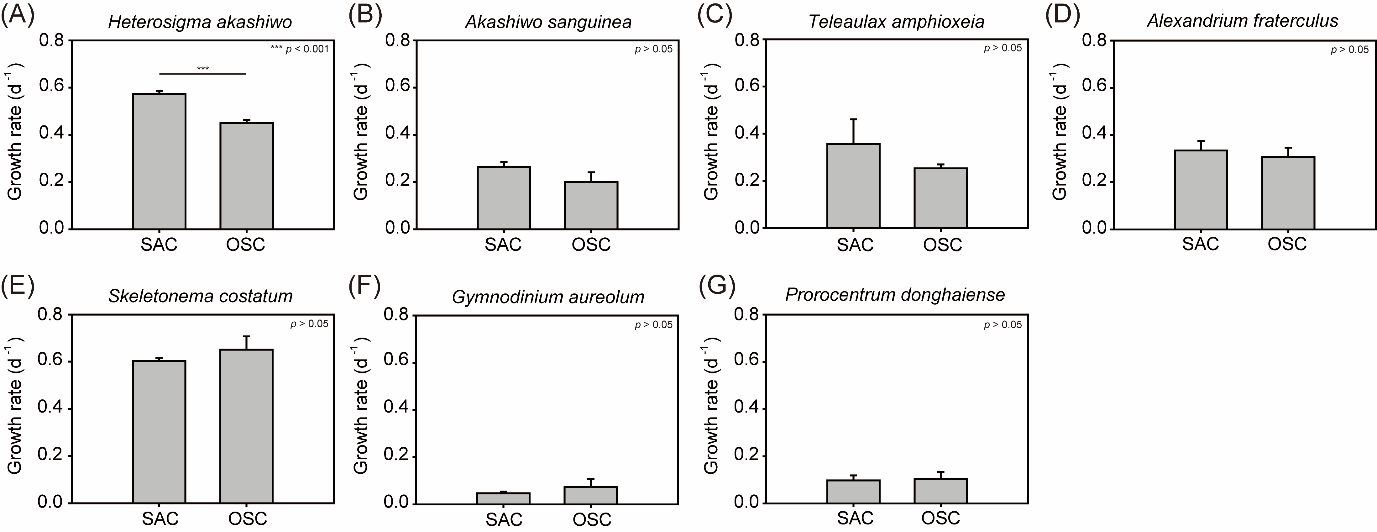


**Supplementary Figure 2.** Growth rate of seven marine phytoplankton in salinity-adjusted control (SAC) and original salinity control (OSC). (A) *Heterosigma akashiwo*, (B) *Akashiwo sanguinea*, (C) *Teleaulax amphioxeia*, (D) *Alexandrium fraterculus*, (E) *Skeletonema costatum*, (F) *Gymnodinium aureolum*, and (G) *Prorocentrum donghaiense*. The *t*-test was performed with n = 3 for each group.

**
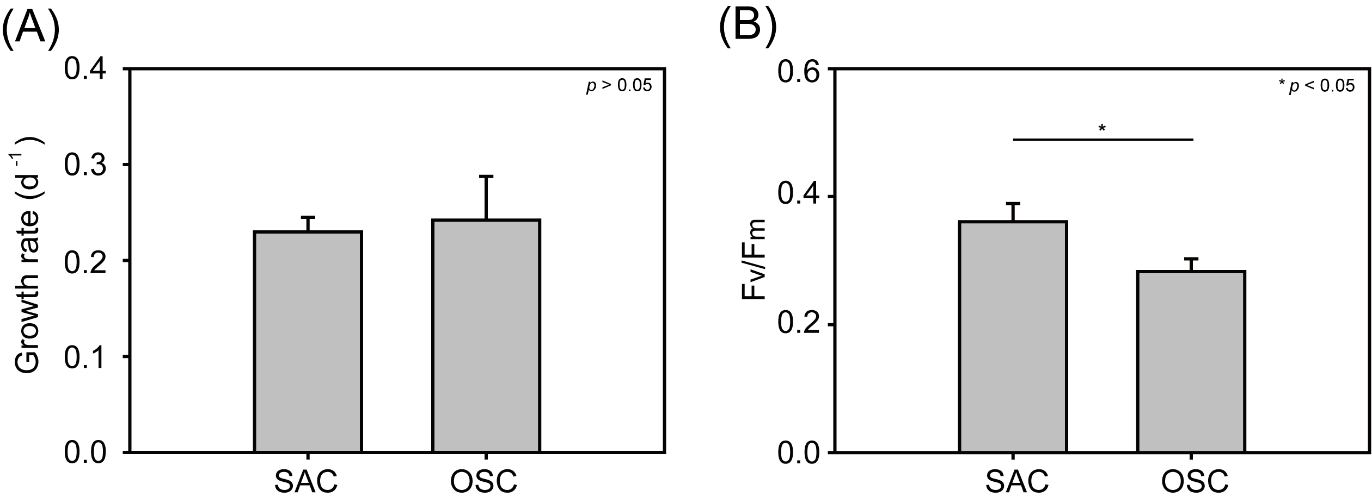
**

**Supplementary Figure 3.** Growth rate (A) and photosynthetic efficiency (B) of *Akashiwo sanguinea* in salinity adjusted control (SAC) and original salinity control (OSC) in Experiment 2. The *t*-test was performed with n = 3 for each group.


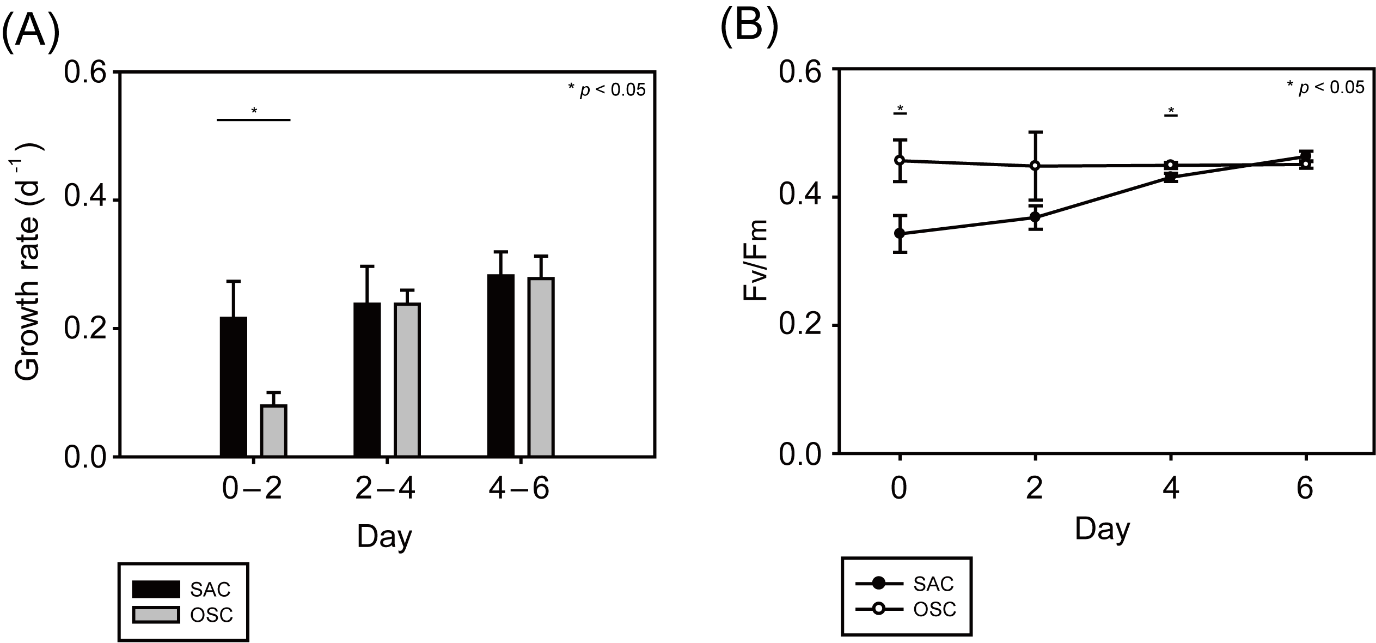


**Supplementary Figure 4.** Growth rate (A) and photosynthetic efficiency (B) of *Akashiwo sanguinea* in salinity adjusted control (SAC) and original salinity control (OSC) during a period of 6 days in Experiment 3. The *t*-test was performed with n = 3 for each group.

**
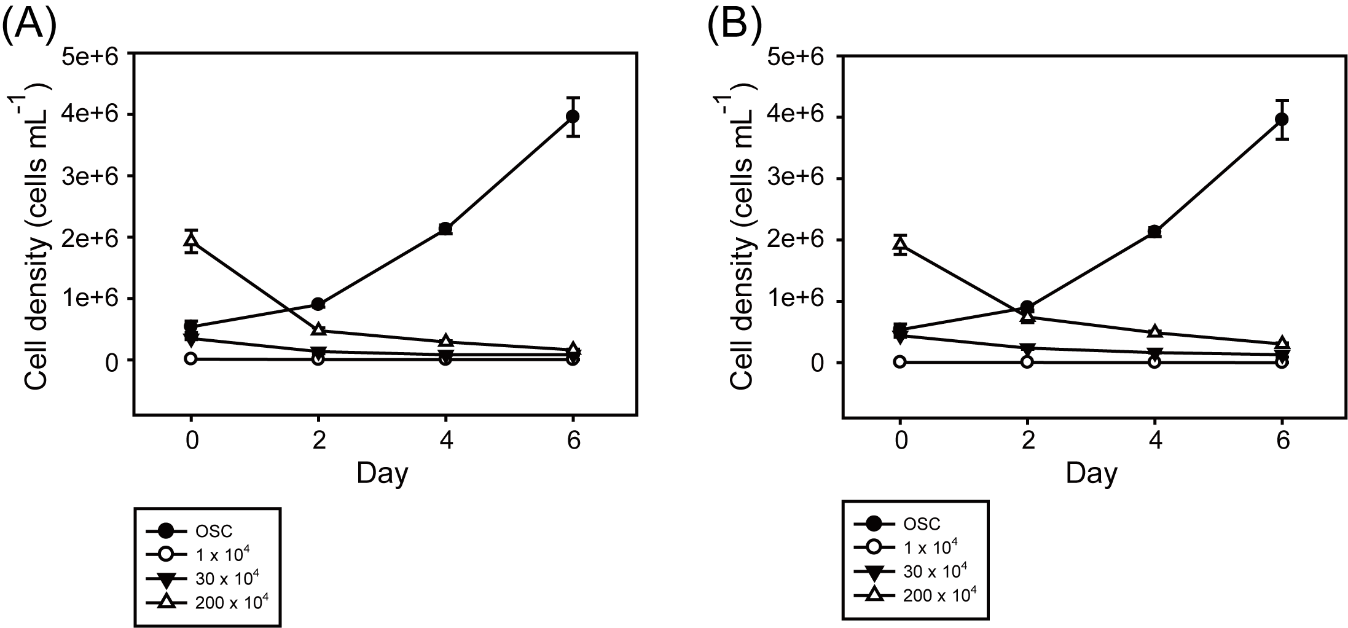
**

**Supplementary Figure 5.** Change in the density of *Microcystis aeruginosa* in the experimental bottles and original salinity control (OSC) (A) and in the control prepared to make all conditions similar with that of the other experimental treatments (CSC) (B) for 6 days. Legends indicate the different concentrations of *M. aeruginosa* in Experiment 3.


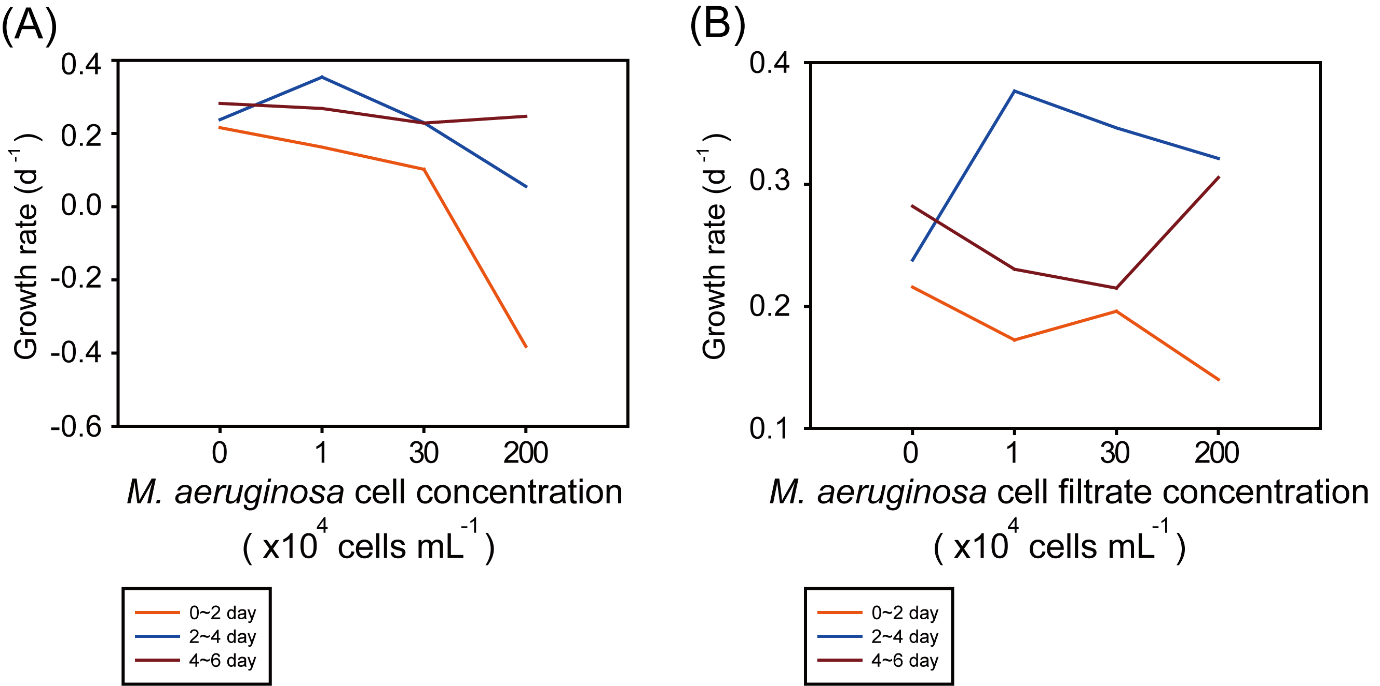


**Supplementary Figure 6.** Profile plot of growth rate of *Akashiwo sanguinea* based on two-way ANOVA analysis when it was co-incubated with *Microcystis aeruginosa* cells (A) and *M. aeruginosa* filtrates (B). Legends indicate the different time points in Experiment 3.

**Supplementary Table 1.** Experimental characteristics of different experiments conducted in the present study. Experiment 1 explored the effects of *Microcystis aeruginosa* on various marine phytoplankton, whereas Experiment 2 and 3 explored the density and time effect of *M. aeruginosa* on *Akashiwo sanguinea,* respectivelyIn addition to *M. aeruginosa* cells, all experiments included equivalent cell-free culture filtrate concentrations of *M. aeruginosa*.

| Group | Species | Initial concentration  of phytoplankton  (cells mL^-1^) | Initial concentration of *M. aeruginosa*  (cells mL^-1^) | Incubation time |
| --- | --- | --- | --- | --- |
| **Experiment 1** |  |  |  |  |
| Dinophyceae | *Akashiwo sanguinea* | 450 | 2,825,200 | 2 days |
| Dinophyceae | *Alexandrium fraterculus* | 1,100 | 1,361,500 | 2 days |
| Dinophyceae | *Gymnodinium aureolum* | 1,800 | 1,374,900 | 2 days |
| Dinophyceae | *Prorocentrum donghaiense* | 3,600 | 1,399,500 | 2 days |
| Raphidophyceae | *Heterosigma akashiwo* | 4,000 | 1,601,300 | 2 days |
| Bacillariophyceae | *Skeletonema costatum* | 4,900 | 1,492,700 | 2 days |
| Cryptophyceae | *Teleaulax amphioxeia* | 6,500 | 1,676,500 | 2 days |
| **Experiment 2** |  |  |  |  |
| Dinophyceae | *Akashiwo sanguinea* | 560/590/580/640/470/590 | 0/9000/30,500/538,600/1,035,700/2,093,100 | 2 days |
| **Experiment 3** |  |  |  |  |
| Dinophyceae | *Akashiwo sanguinea* | 490/520/510/520 | 0/11,200/348,000/1,931,000 | 6 days |

**Supplementary Table 2.** Summary of two-way ANOVA analysis on the effect of *Microcystis aeruginosa* cell (C to C) and filtrate (C to F) on the growth rate (A) and photosynthetic efficiency (B) of *Akawhiso sanguinea*. The *p*-values obtained from the two-way ANOVA were adjusted using the Bonferroni correction. Sample size for each group is n = 3.

(A)

|  | Growth rate of *A. sanguinea* (C to C) | | | | | |  | Growth rate of *A. sanguinea* (C to F) | | | | | |
| --- | --- | --- | --- | --- | --- | --- | --- | --- | --- | --- | --- | --- | --- |
| Factor | df | Sum of squares | Mean squares | F value | *p*-value | *η*_p_ ^2^ | | df | Sum of squares | Mean squares | F value | *p*-value | *η*_p_ ^2^ |
| Concentration | 3 | 0.476 | 0.159 | 48.643 | 0.000 | 0.859 | | 3 | 0.002 | 0.001 | 0.460 | 1.000 | 0.054 |
| Time | 2 | 0.372 | 0.186 | 56.927 | 0.000 | 0.826 | | 2 | 0.103 | 0.052 | 30.730 | 0.000 | 0.719 |
| Concentration-time Interaction | 6 | 0.345 | 0.058 | 17.632 | 0.000 | 0.815 | | 6 | 0.050 | 0.008 | 4.935 | 0.006 | 0.552 |

(B)

|  | Photosynthetic efficiency of *A. sanguinea* (C to C) | | | | | |  | Photosynthetic efficiency of *A. sanguinea* (C to F) | | | | | |
| --- | --- | --- | --- | --- | --- | --- | --- | --- | --- | --- | --- | --- | --- |
| Factor | df | Sum of squares | Mean squares | F value | *p*-value | *η*_p_ ^2^ | | df | Sum of squares | Mean squares | F value | *p*-value | *η*_p_ ^2^ |
| Concentration | 3 | 0.816 | 0.272 | 276.210 | 0.000 | 0.963 | | 3 | 0.013 | 0.004 | 3.502 | 0.081 | 0.247 |
| Time | 3 | 0.131 | 0.044 | 44.239 | 0.000 | 0.806 | | 3 | 0.083 | 0.028 | 22.634 | 0.000 | 0.680 |
| Concentration-time interaction | 9 | 0.088 | 0.010 | 9.870 | 0.000 | 0.735 | | 9 | 0.019 | 0.002 | 1.694 | 0.396 | 0.323 |
